# Analyzing the Effect of Particle Shape on Deformation Mechanism during Cutting Simulation of SiC p/Al Composites

**DOI:** 10.3390/mi12080953

**Published:** 2021-08-12

**Authors:** Jiakang Zhou, Jieqiong Lin, Mingming Lu, Xian Jing, Yubo Jin, Dunlan Song

**Affiliations:** School of Mechatronic Engineering, Changchun University of Technology, Changchun 130012, China; zhoujiakang07@163.com (J.Z.); jingxian@ccut.edu.cn (X.J.); jyb1245927658@163.com (Y.J.); songdunlan@ccut.edu.cn (D.S.)

**Keywords:** metal matrix composites, machinability, finite element, deformation mechanism

## Abstract

To analyze the effect of particle shape on deformational behavior in the cutting simulation process for metal matrix composites (MMCs), two 2D mesoscopic-based finite element (FE) models reinforced with randomly distributed circular and irregular polygonal particles were developed. Different material properties (metal matrix phase, particle reinforced phase) and the properties of the particle–matrix interface were comprehensively considered in the proposed FE model. Systematic cutting experiments were conducted to compare the differences between two modeling approaches with respect to particle fracture, chip formation, cutting force and surface integrity. The results show that the irregular polygonal particle model is closer to the microstructure of MMCs, and is better able to reflect the deformation behavior of particles. The simulation model with irregular polygonal particles is even able to capture more details of the impact caused by particles, reflecting variations in the cutting force in the actual cutting process. The initiation and propagation of microcracks is mainly determined on the basis of particle geometry and further affects chip formation. Both models are able to correctly reflect surface defects, but the irregular polygonal particle model provides a more comprehensive prediction for the subsurface damage of MMCs.

## 1. Introduction

Traditional metal materials are gradually being replaced by metal matrix composites (MMCs) in the fields of aerospace, automobiles, optical instruments and electronic packaging due to their excellent material properties, which include high specific stiffness, high modulus, greater wear resistance, etc. [1,2,3]. In general, MMCs are made by combining ceramic-reinforced particles and metal matrix using physical methods, resulting in material properties that are better than any single-phase material. However, the machinability of MMCs is poor, as the addition of reinforcements such as ceramic particles makes MMCs difficult to cut [4]. In addition, the mechanical properties of MMCs are also affected by the geometric parameters of the reinforcing phase, such as the aspect ratios of TiB whiskers formed in situ [5] and the length of carbon nanotubes [6]. Therefore, research into deformation behaviors is significant for improving the machinability of MMCs.

At present, the research on the deformation mechanisms of MMCs is mainly realized on the basis of experiments and simulations. With respect to the material deformation behavior of MMCs under extreme conditions, Zherebtsov et al. [7,8,9] researched the effects of the microstructure and mechanical properties of MMCs during high-temperature deformation and high-pressure torsion. Meanwhile, with respect to the mechanism of cutting deformation, the experimental method is able to objectively reflect the processing state of MMCs, including cutting force, tool wear, chip morphology and surface quality [10,11,12]. Nevertheless, the further development of experimental methods is further limited by the huge processing costs and time consumption. With the rapid development of computer technology, the finite element (FE) method has attracted increasing attention from scholars [13,14,15]. Compared with experimental methods, the FE method is able to reveal the cutting process directly and in detail, which is of great significance for analyzing chip formation, matrix and particle fracture, subsurface damage, etc.

Zhu et al. [16] established an FE model for alumina/aluminium 6061 MMCs based on plane-strain thermo-elasto-plastic with particles of random shape and position. In their model, the particle–matrix interface was set to have the same hardness as alumina, and the interface thickness was defined as 1 μm. In addition to the stress–strain distribution and the interface temperature, the results also showed that the scratch of the rake face was mainly caused by alumina particles. Pramanik et al. [17] analyzed the workpiece deformation and the tool–particle–matrix interactions in the orthogonal cutting process of MMCs, considering the relative position of the particles and the cutting edge (i.e., with particles along, above and beneath the cutting path, respectively). Circular reinforcements were inclined towards the cutting direction in the matrix in order to characterize the different positional relationships between the particles and the tool. However, they did not consider particle fracture for the facilitation of the modeling and calculation process. Zhou et al. [18] proposed an equivalent homogeneous model and a multi-phase microscopic FE model of SiC p/Al composites with a 56% volume fraction. The multi-phase microscopic FE model was applied to study the removal mechanism and stress distribution of SiC particles without considering the distribution and dimensions of the particles or the particle–matrix interface. Subsequently, Zhou et al. [19,20] investigated the formation mechanism of edge defects in the cutting process of SiC p/Al composites, establishing a multi-particle microscopic FE model that was reinforced by irregular polygonal particles with a random distribution. The results showed that the surface quality and edge defects were mainly determined by the fracture forms of the SiC particles, including crushing, cutting through, fracture and pulling out from the metal matrix. In 2015, Wang et al. [21] established two 2D meso-scale FE models of SiC p/Al composites reinforced by randomly distributed circular and polygonal particles in order to study the formation mechanism of surface defects in the milling process. The effects of particle fracture and un-fracture on the simulation results were analyzed by varying the particle properties. It was shown that the rotation and displacement of the SiC particles, pits, microcracks and ploughing effect were the main forms of surface defects. However, the effect of particle shape on chip formation, cutting force and particle deformation behavior were not analyzed in detail.

Compared with the high brittleness and high volume fraction of MMCs, the plastic deformation of Al and the fracture of SiC particles dominate chip formation during the cutting process of low-volume-fraction MMCs. To investigate particle fracture and debonding, as well as tool–particle interaction, Ghandehariun et al. [22] developed a micro-mechanical FE model of Al6061 reinforced by Al_2_O_3_ particles with uniform distribution. Alumina particles were regarded as completely elastic materials, and their fracture process was described using a brittle fracture model. Nevertheless, there are some differences between the uniformly distributed reinforced particles and the actual microstructure of MMCs [23]. Teng et al. [24,25] established FE models of MMCs reinforced by nano- and microparticles, respectively. Although these were able to reflect the stress distribution during the material deformation of the workpiece to a certain extent, the gradient of stress distribution was greatly affected by the distribution of particles. Compared with the uniform distribution, a random distribution of particles can reflect the internal microstructure of MMCs to the greatest extent, thus reducing the impact of modeling error on the simulation process. To validate the three-phase friction model of MMCs, Duan et al. incorporated the improved frictional coefficient into the FE model with randomly distributed round particles of SiC p/Al composites [26]. They verified three types of friction at the tool–chip interface (two-body sliding friction, three-body rolling friction and matrix-rake face friction) and the formation mechanism of serrated chips. However, particle fracture was not considered in this model, and a detailed description of the modeling process was lacking. The fracture behavior of SiC particles was considered in the FE model established by Laghari et al. [27]. A brittle fracture material model was used to characterize the failure behavior of SiC particles. The SiC particles in their model had an elliptical geometry and were randomly distributed in the cutting layer. They analyzed the stress distribution of a single SiC particle in different cutting paths, which reflected particle–tool interaction, but ignored the interaction between particles. The same situation can be seen in the study of Wu et al. [28], who analyzed the interaction between a single circular SiC particle and the tool tip.

Recently, irregular polygonal particles have gradually begun to be applied for the modeling of MMCs [29]. To study the chip formation mechanism and subsurface damage, Wu et al. [30,31] established 2D and 3D microstructure FE models of Al359/SiC composites by comprehensively considering the dimensions and distribution of particles, the debonding of the particle–matrix interface, and the fracture of the matrix and particles. They converted microstructure images of MMCs into binary images, and described the practical structure on the basis of the image processing. Yu et al. [32] employed the Johnson–Holmquist (JH-2) ceramic material model to characterize the properties of SiC particles, and studied the removal mechanism and damage behavior in detail. Discontinuous chips were easier to separate from the workpiece, where particles accumulate, and the stress concentration in the corners of SiC particles was more serious.

The above research shows that different simulation models are able to simulate the deformation process of MMCs to a certain extent. Circular particles can simplify the modeling process and reduce the calculation cost, while irregular particles are closer to the actual microstructure of MMCs, but also present higher requirements for the modeling process. Therefore, in order to research the influence of particle shape on the simulation process, it is necessary to analyze the influence of different particle geometries on deformational behavior in MMC cutting simulation, as well as the consistency between the simulation results and the experimental results.

In this paper, two 2D mesoscopic-based FE models of MMCs reinforced with randomly distributed circular and irregular polygonal particles are developed to compare the influence of particle shape on deformational behavior in the MMC cutting simulation process. Matrix deformation behaviors, the distribution and fracture of particles, and the interaction at matrix–particle interfaces will be comprehensively considered in the proposed FE models. Finally, systematic experiments are executed to validate the effect of particle shape on deformation mechanism during cutting simulation of MMCs.

## 2. Finite Element Modeling Procedure

### 2.1. Model Descriptions

**Figure 1:**
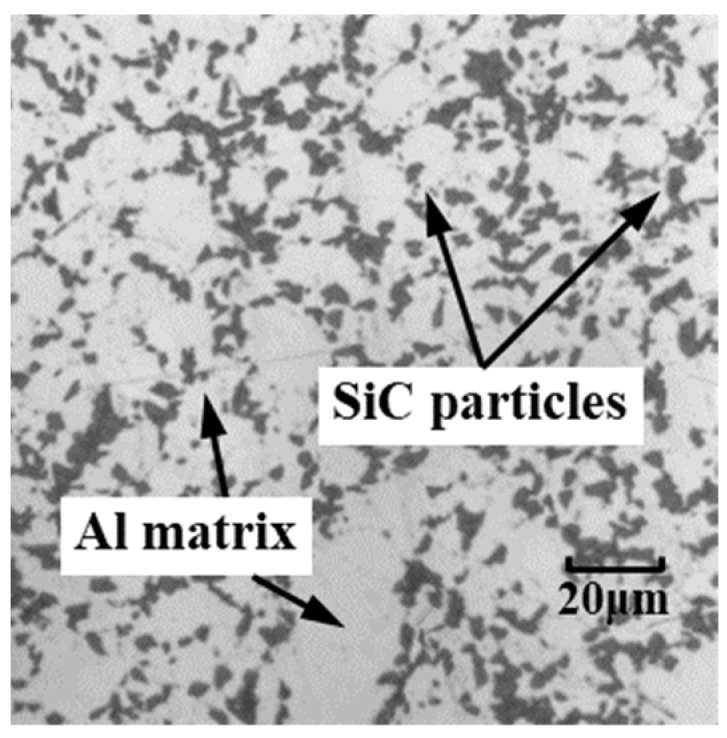
Microstructure of the SiC p/Al composite.

**Figure 2:**
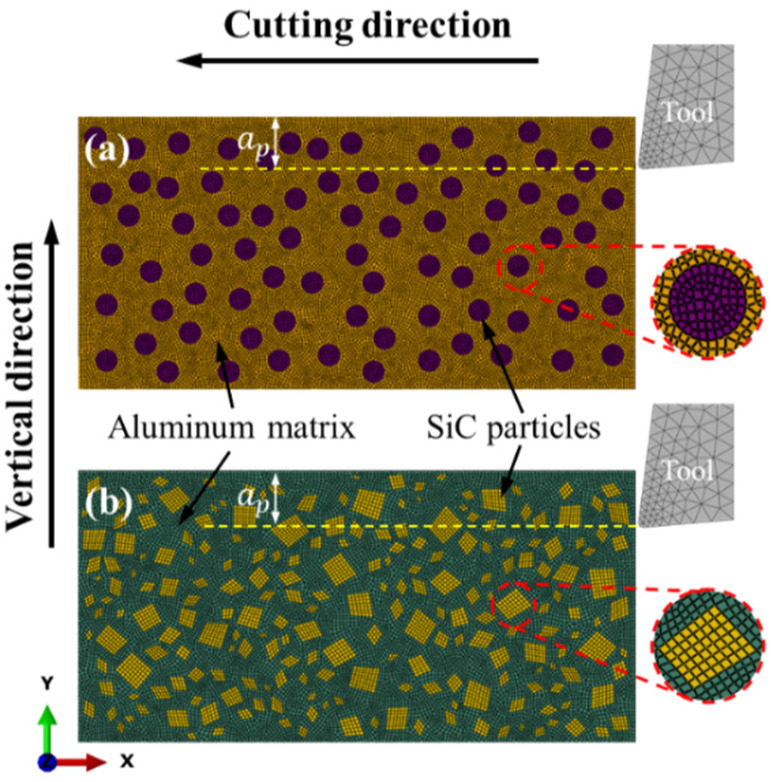
2D mesoscopic-based finite element (FE) model setup for orthogonal SiC p/Al composites reinforced with different particle shapes: (**a**) circular particles; (**b**) irregular polygonal particles.

To simulate the cutting process of MMCs, SiC p/Al composites with a 25% volume fraction of reinforcements are applied as the sample material, with an average particle size of 15 μm. The microscope image of SiC p/Al composites is shown in Figure 1. The two 2D mesoscopic-based FE models setup for orthogonal SiC p/Al composites reinforced with circular and irregular polygonal particles based on ABAQUS/CAE 2020 are shown in Figure 2. The dimensions of the FE models were set as 0.5 mm in length and 0.25 mm in width, and the sizes of the particles were set as 15 μm for the circular and 5–20 μm for the irregular polygons to characterize the microstructure of the SiC p/Al composites.

The free meshing technique was employed to determine the mesh properties in FE models of SiC p/Al composites, and four-node bilinear plane stress quadrilateral elements (CPS4R) were used to control the element type. To increase the accuracy of the simulation process, the minimum sizes of single elements in the metal matrix and SiC particles were 1.5 µm and 1 µm, respectively, as shown in Figure 2. A mass scaling option was applied to improve the computational efficiency. The global insert cohesive method was used by means of a professional plug-in to describe the interface of elements. The tool was assumed to be a rigid body, with a 7° rake angle and a 5° flank angle. To reduce the influence of the machining parameters on the simulation results, a constant cutting speed of 250 mm/s and a depth of cut of 25 μm were used in the simulation model. The specific machining parameters and tool specifications are shown in Table 1.

Both workpieces were constrained against moving in any direction at the bottom and left-hand side in the FE models. The cohesive section and damage initiation for the cohesive elements were determined on the basis of the traction separation law and the maximum nominal stress criterion. In addition, the failure evolution behavior of the cohesive elements was described by energy approach.

### 2.2. Material Properties of Aluminum Matrix

The Johnson–Cook (JC) constitutive model is usually adopted to simulate the deformation process of materials under dynamic loading [33]. As a typical deformable thermoelastic–plastic material, the JC constitutive model is used to characterize the constitutive model of the aluminum matrix in this study, which can be expressed as follows:

(1) $$\sigma =\left[A+B\varepsilon^{n}\right]\left[1+C\ln\left(\frac{\dot{\varepsilon }}{{\dot{\varepsilon }}_{0}}\right)\right]\left[1-{\left(\frac{T-T_{r}}{T_{m}-T_{r}}\right)}^{m}\right]$$

The equation represents the relationship between stress, strain rate and temperature in the process of deformation. Because only the cutting deformation process of MMCs is analyzed, increases in temperature were not considered in this study. In addition, the material parameters *A*, *B*, *C* and *n* can be calculated using the Hopkinson bar tensile test [26].

The JC damage criterion considers stress triaxiality, the strain rate and the temperature effect, and possesses a more extensive adaptability [34]. Therefore, the JC damage criterion is used to describe the failure behavior of the metal matrix. The element damage behavior characterized by the JC damage criterion is shown below:

(2) $$D=\sum \frac{\Delta \varepsilon }{\varepsilon^{f}}$$

where *D* represents the damage parameter, with a value of 0–1, which can be expressed as the initial state and element damage, respectively. Δε is the strain increment of plastic during a single time step, and $\varepsilon^{f}$ represents the failure strain under the stress state, the strain rate, and the failure strain at the current time step, which can be represented as follows:

(3) $$\varepsilon^{f}=\left(D_{1}+D_{2}\exp\left(D_{3}\sigma *\right)\right)\left(1+D_{4}\ln\dot{\varepsilon }\right)\left(1+D_{5}T*\right)$$

where *D*_1_, *D*_2_, *D*_3_, *D*_4_ and *D*_5_ represent the material parameters of the metal matrix. In Equation (3), the first term is the correlation function of pressure, equivalent stress and stress triaxiality, the second term is the plastic strain rate, and the third term is dimensionless temperature. The material properties and JC constants of Al are listed in Table 2.

### 2.3. Material Properties of SiC Particles

**Figure 3:**
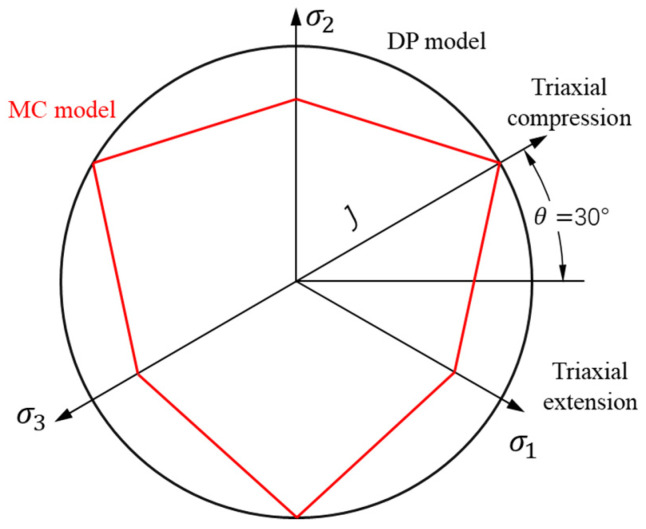
Drucker–Prager (DP) and Mohr–Coulomb (MC) models in the deviatoric plane.

As an elastic–plastic model, when the deformation of the material reaches the yield limit of SiC, the stress does not change, while the strain increases constantly. The deformation behavior of SiC particles can be characterized using the Drucker–Prager (DP) model. The DP model considers the effect of intermediate principal stress on the yield and fracture of the material, and amends the singularity caused by the sharp corners in the Mohr–Coulomb (MC) model [35]. The corner of the yield surface of the DP model is smooth and conical in the principal stress space, as shown in Figure 3. The DP model is expressed as follows:

(4) $$f\left(I_{1},\sqrt{J_{2}}\right)=\sqrt{J_{2}}-aI_{1}-k=0$$

where the constants *a* and *k* are related to the cohesion *c* and the internal friction angle of materials ϕ. $I_{1}$ is the first invariant of the stress tensor, and $J_{2}$ is the second invariant of the stress partial tensor, which can be represented as follows:

(5) $$a=\frac{2\sin\phi }{\sqrt{3}\left(3-\sin\phi \right)}$$

(6) $$k=\frac{3-\sin\phi }{3+\sin\phi }$$

(7) $$I_{1}=\sigma_{1}+\sigma_{2}+\sigma_{3}$$

(8) $$J_{2}=\frac{1}{6}\left[{\left(\sigma_{1}-\sigma_{2}\right)}^{2}+{\left(\sigma_{2}-\sigma_{3}\right)}^{2}+{\left(\sigma_{3}-\sigma_{1}\right)}^{2}\right]$$

In addition, the maximum normal stress fatigue criterion is adopted to describe the fracture behavior of the SiC particles in this study. When the external force acting on an element exceeds the critical value of fracture stress, brittle fracture will occur along interface where the maximum tensile stress is located. The maximum tensile stress $\sigma_{0}$ is applied to account for the brittle fracture of material, and can be expressed as follows:

(9) $$\max\left(\sigma_{1},\sigma_{2},\sigma_{3}\right)=\sigma_{0}$$

where $\sigma_{1},\sigma_{2}$ and $\sigma_{3}$ represent components of principal stress acting on the element. Crack propagation behavior is determined by the Mode II fracture mode, while the crack propagation modulus $G_{c}$ is determined as shown below:

(10) $$G_{c}={\left(1-\frac{e^{ck}}{e_{\max}^{ck}}\right)}^{p}G$$

where $e^{cr}$ is fracture strain. *G* represents shear modulus of unfractured particles. *p* and $e_{\max}^{ck}$ represent material parameters controlling shear retention, which were obtained on the basis of the tensile force displacement curve of the SiC material [36]. The material properties and constants of SiC particles are listed in Table 3.

### 2.4. Properties of the Particle–Matrix Interface

**Figure 4:**
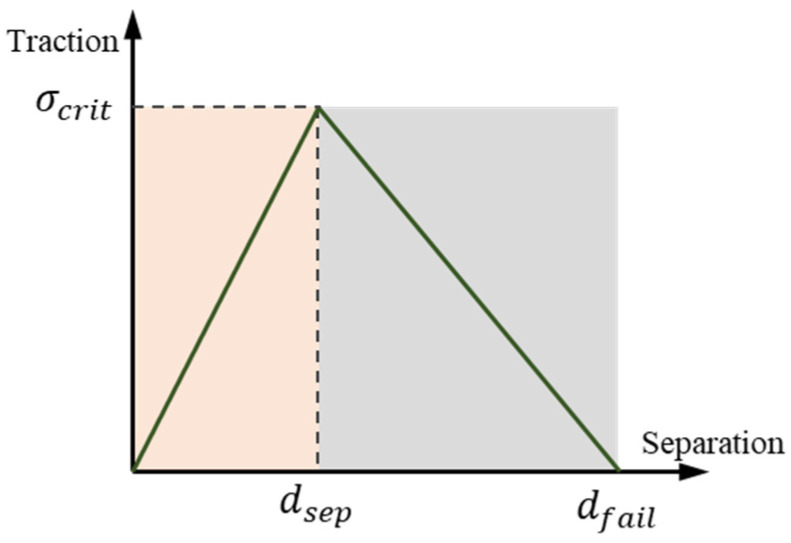
Typical traction–separation response.

The cohesive zone model (CZM) has been successfully applied on the machining simulation process of carbon fiber-reinforced composites (CFRP) to predict the debonding phenomena at the fiber–matrix interface [37,38]. As shown in Figure 4, CZM reveals a relationship between the interfacial traction force and initial crack displacement. Under continuous traction, two contact surfaces begin to separate, and traction force first increases and then decreases with increasing separation distance based on the traction separation law [39]. When a separation displacement $d_{sep}$ causes the traction stress between two contact surfaces to reach the critical value of $\sigma_{crit}$, the cohesive elements begin to fail; when the separation distance reaches a certain value of $d_{fail}$, the interface completely separates and debonds. This characteristic of cohesive elements enables CZM to evaluate the debonding process at the particle–matrix interface during the simulation process.

The traction separation law can be expressed as follows:

(11) $$F\left(\xi \right)=\frac{27}{4}\sigma_{\max}\left(1-2\xi +\xi^{2}\right)$$

where $\sigma_{\max}$ represents the cohesive strength. Another important parameter of the traction separation law is the normal separation energy *Gc*, which can be indicated by the area under the traction–separation response curve [40].

To define the damage initiation of CZM, a maximum nominal stress criterion is used to account for the damage behavior of the cohesive element, as given below:

(12) $$\xi =\left\{\frac{\langle t_{n}\rangle }{t_{n}^{0}},\frac{t_{s}}{t_{s}^{0}},\frac{t_{t}}{t_{t}^{0}}\right\}=1$$

where the dimensional parameter ξ is determined by the normal ($t_{n}$) and tangential ($t_{s}$ and $t_{t}$) component, and the maximum allowable normal ($t_{n}^{0}$) and tangential ($t_{s}^{0},t_{t}^{0}$) component of the cohesive element. Damage occurs when the dimensional parameter of the cohesive element ξ reaches 1. The damage evolution at the particle–matrix interface is related to the interface properties, as illustrated in Table 4 [21].

### 2.5. Interaction

The interaction of the tool and the workpiece is defined by surface-to-surface contact. The rake face, the cutting edge and the flank face of the tool are set as the master surface, and the chip layer node is set as the slave surface. To reduce the influence of the discretization mode of the master and slave surfaces in the process of material deformation, the slave surface element is smaller than the master surface element (the minimum mesh size of the workpiece is 1.5 μm). The Coulomb friction model is adopted to describe the contact friction and stick–slip state between the tool and the workpiece:

(13) $$\left\{ \begin{array}{ll} \tau =\tau_{\max} & \mu \sigma_{n}<\tau_{\lim}(stick)\\ \tau =\mu \sigma_{n} & \mu \sigma_{n}\geq \tau_{\lim}\;(slip)\; \end{array}\right.$$

The relative positions of the tool and the workpiece remain unchanged in the stick state, but the normal compressive stress $\sigma_{n}$ induced by extrusion of the rake face produces a shear stress at the rake face–chip bottom interface that is less than the interfacial shear stress limit $\tau_{\lim}$ (a function of normal compressive stress $\sigma_{n}$ and friction coefficient μ). The relative slip of interface occurs when the shear stress exceeds the interfacial shear stress limit $\tau_{\lim}$. The contact state is dominated by the friction coefficient μ of the contact surfaces between the rake face and the chip bottom. Considering the influence of multiphase materials, the friction coefficient μ is defined by the three-phase friction model proposed by Duan et al. [26].

## 3. Experimental Conditions

**Figure 5:**
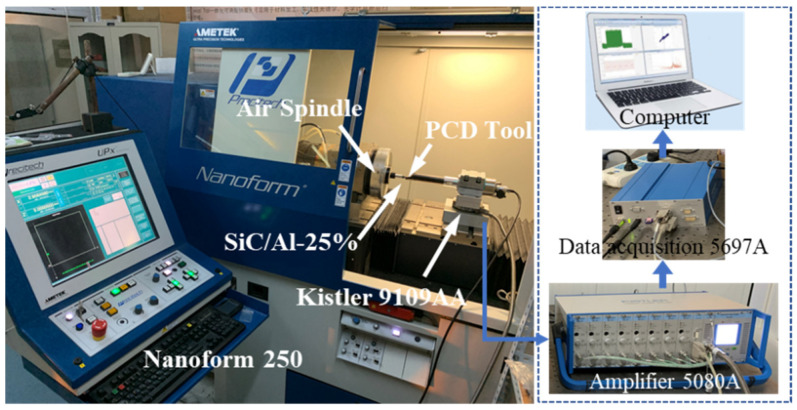
Experimental setup.

The validation experiments were carried out using a Precitech Nanoform 250 CNC precision lathe machine (AMETEK^®^ Precitech, Inc., Keene, NH, USA). The material sample was 12.7 mm in diameter and 10 mm in length, with a 25% volume fraction of SiC particles. The geometrical parameters of the practical tool and the cutting experiment were same as those in the simulation model. During the cutting experiments, a dynamometer 9109AA provided by Kistler (Winterthur, Switzerland) was used to measure the actual cutting force. Chip morphology and surface integrity were measured by white light interferometer (Zygo NewView 8000, Zygo Corporation, Berwyn, PA, USA). The experimental setup is shown in Figure 5.

## 4. Results and Discussion

### 4.1. Particle Deformation Behavior

**Figure 6:**
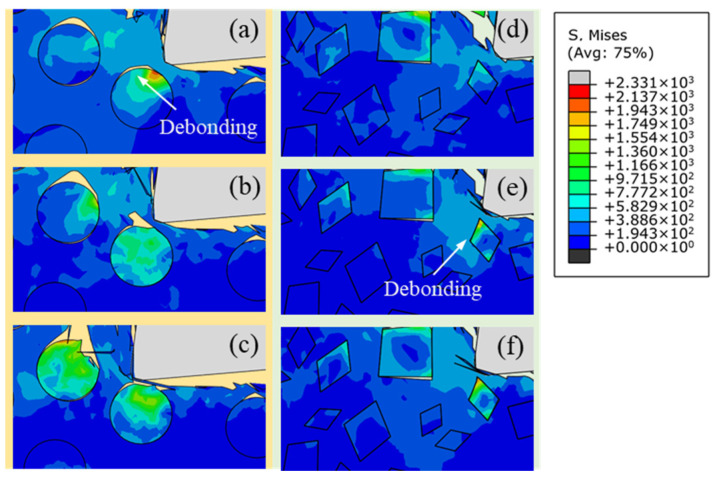
Stress distribution of particles on the cutting path with different particle shapes: (**a**–**c**) circular particles; (**d**–**f**) irregular polygonal particles.

Figure 6 presents the stress distribution of particles with different shapes on the cutting path at a cutting speed of 250 mm/s and a cutting depth of 25 μm. Plastic deformation of the metal matrix occurs with the extrusion of the rake face. Meanwhile, severe stress concentration phenomena also appear at the particle–matrix interface. As the tensile strength of SiC particles is greater than the bonding strength of the interface, interface debonding occurs with the advancement of the tool until the tool comes into direct contact with the particle, and the tensile stress on both sides of interface exceeds the bonding strength of the interface. For circular particles, the compressive stress in particles rises sharply ahead of the tool tip (Figure 6a). With the crushing of the tool into the workpiece, metal matrix flows along the rake face with the squeezing of the rake face, causing the tensile stress at the particle–matrix interface to increase rapidly until the interface fails, as shown in Figure 6b,c. Interface debonding is indirectly caused by the tool–particle interaction transmitted by the metal matrix. However, the stress concentration area of irregular polygonal particles is not only affected by tool extrusion, but also the interaction of the surrounding particles due to the presence of sharp corners. As shown in Figure 6d,e, stress concentrations appear on the sides of polygonal particles far away from the tool tip, with debonding subsequently occurring at the particle–matrix interface. With the advancement of the tool, the SiC particles fracture when the interaction of the particle and the tool exceeds the tensile strength of the particle (Figure 6f). Moreover, fractured particles move and rotate in metal matrix driven by the cutting edge of the tool, leading to particles being partially or completely detached from the metal matrix, severe subsurface damage, and large cavities.

**Figure 7:**
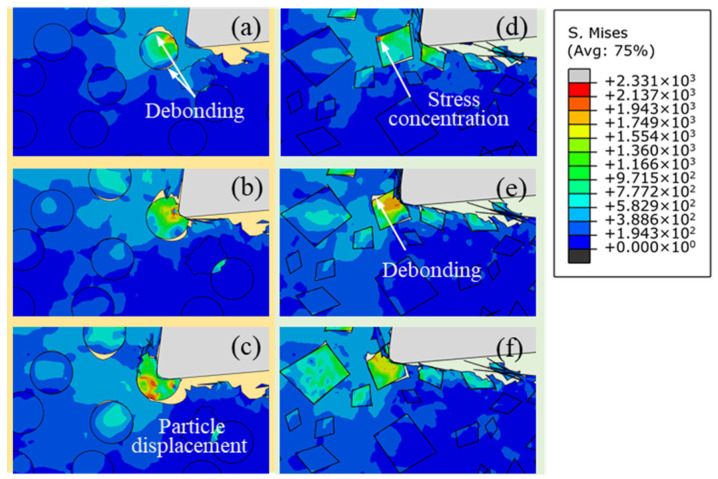
Stress distribution of particles below the cutting path with different particle shapes: (**a**–**c**) circular particles; (**d**–**f**) irregular polygonal particles.

**Figure 8:**
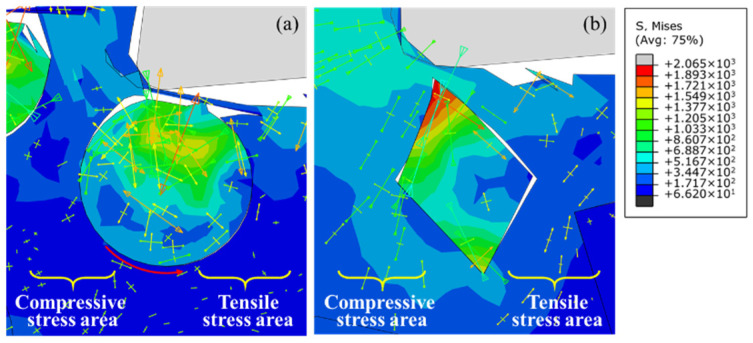
Stress distribution of single particles below the cutting path with different particle shapes: (**a**) circular particles; (**b**) irregular polygonal particles.

Figure 7 is the stress distribution of particles with different shapes below the cutting path at a cutting speed of 250 mm/s and a depth of cut of 25 μm. The metal matrix between the cutting edge and the SiC particles is still responsible for the transfer of the interaction between the tool and the particles, as shown in Figure 7a,d. The stress caused by the non-uniform plastic deformation of metal matrix in the third deformation zone is transferred to the upper part of a particle when the tool approaches the particle, and stress concentration occurs under the influence of the particle–tool interaction. Interfacial debonding mainly occurs in the part of the particle near the cutting edge, as shown in Figure 7b,e. On the other hand, the stress is far less than the tensile strength of the SiC particle at this moment, so no particle fracture will take place, as shown in Figure 7c,f. The simulation results show that stress concentration near the cutting edge and particle–matrix interface debonding phenomenon demonstrate good consistency with different particle shapes. However, the distribution of stress on irregular polygonal particles is approximately centrosymmetric. Figure 8 shows the stress distribution of single particles below the cutting path with particles with different shapes. It is clear that the stress distribution of irregular polygonal particles is determined by the elastic–plastic deformation of the metal matrix on both sides of the particles. The metal matrix on both sides of the particles is affected by compressive stress from the cutting edge and tensile stress from the elastic recovery of the machined surface. Due to the geometry of the particles, it is easy for the stress to bypass the particle–matrix interface and be transferred to the other side of the particle, as shown in Figure 8a; meanwhile, the stress will be hindered and accumulate at the sharp corners of irregular polygonal particle, thus forming stress concentration, as shown in Figure 8b. In contrast to the particle–matrix interface, which plays a role of uniform transition in circular particle model, the particle–matrix interface in the irregular polygonal particle model is not able to uniformly transfer the stress to both sides of particles under the trend of particle rotation, leading to a centrosymmetric stress distribution in particles.

**Figure 9:**
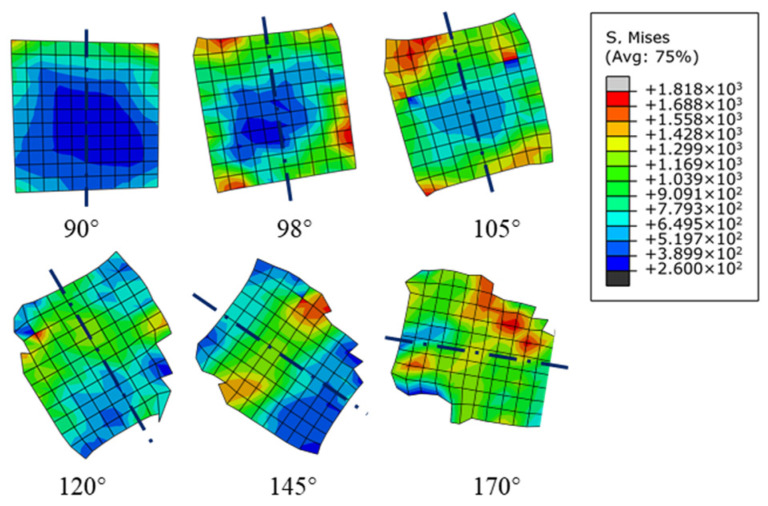
Particle rotation during cutting simulation of SiC p/Al reinforced with irregular polygonal particles.

Figure 9 presents the particle rotation during the cutting simulation of SiC p/Al composites reinforced with irregular polygonal particles above the cutting path. With the advancement of the tool, the friction between the rake face and the metal matrix causes chips to formed from the broken matrix, flowing upward along rake face. The particle–matrix interface near the rake face of the tool starts to fail, leading differences in the speed of plastic deformation on both sides of the particles. Great differences in stress between different sides of the particles can promote the generation of differences in speed, resulting in the particles rotating anticlockwise, as illustrated in Figure 9. However, areas of concentrated stress are not easily produced around circular particles, as there are no sharp corners, and the differences in stress that could promote the rotation of the particles cannot be formed on both sides of the particle.

### 4.2. Cutting Force

**Figure 10:**
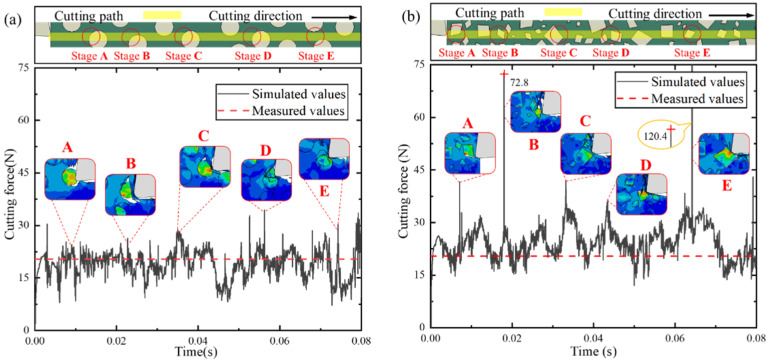
Cutting force variation under a cutting speed of 250 mm/s and a cutting depth of 25 μm with different particle shapes: (**a**) circular particles; (**b**) irregular polygonal particles.

Variations in cutting force under a cutting speed of 250 mm/s and a cutting depth of 25 μm with circular and irregular polygonal particles are shown in Figure 10. Due to the limitations of the natural frequency of the dynamometer (15 kHz) and the sampling frequency, average values of multiple measurements of the cutting force were used in order to reduce measurement error.

As shown in Figure 10, the models with both circular and irregular polygonal particles were able to reflect changes in the cutting force during the cutting simulation process, and the variation curve vibrates at close to the experimental values, indicating that the simulation models are in good agreement with the actual cutting process. In addition, the variation curve of the cutting force is characterized by large fluctuations and small fluctuations. Large fluctuations are dominated by the impact of the reinforced particles on the rake face and the continuous change of the contact area caused by the segmented chips. In addition, the complicated friction state of the rake face–chip bottom interface (three-body rolling friction and two-body sliding friction) and the change of stress distribution of the slave surface induced by the setting of the interface properties in the simulation model are able to explain the small fluctuations. However, the cutting force obtained by the circular particle model is closer to the experimental value, while the value acquired using the irregular polygonal particle model is greater than the experimental value. It may be that the greater number of particles in the irregular polygon model increase the probability of contact between the particles and the rake face of the tool under the same random distribution criterion and volume fraction ratio of SiC p/Al composites. Moreover, the variation curve of the force values increases abruptly with the irregular polygonal particle model, while the cutting force curve of the circular particle model is more stable, which can be explained by the stress concentration in the particles on the cutting path, especially at sharp corners of the irregular polygonal particles.

In general, the FE model reinforced by irregular polygonal particles is able to characterize the actual microstructure of SiC p/Al composites, as shown in Figure 1 and Figure 2b. As a result of the natural frequency of the dynamometer mentioned above, the dynamometer may not be able to capture every impact of every SiC particle on the rake face during the cutting experiment, so the measured values will be reduced to a certain extent. Therefore, the irregular polygonal particle model is more reasonable for reflecting changes in the cutting force during the actual cutting process of SiC p/Al composites than the circular particle model. The same conclusion was found in research proposed by Wang et al. [21]. They pointed out that the main reason the cutting force of MMCs was greater than that of homogeneous materials was that the SiC particles located on the cutting path come into contact with each other and hinder the advancement of the tool. In addition, serious concentrations of stress also appeared as a result of the sharp corners of the irregular polygonal particles.

### 4.3. Chip Formation and Morphology

In the cutting process of MMCs, the formation, propagation and convergence of microcracks has a significant impact on chip formation and morphology, particle fracture and debonding, and surface integrity.

**Figure 11:**
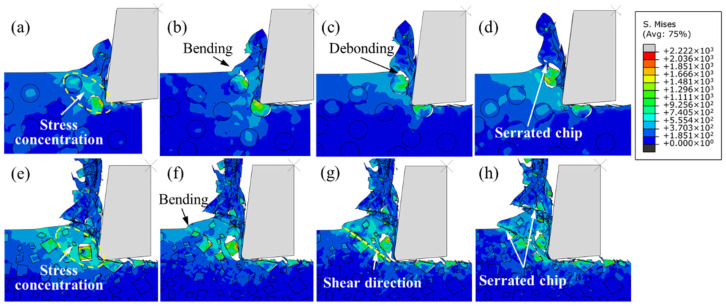
Mises stress distribution during chip formation: (**a**–**d**) circular particles; (**e**–**h**) irregular polygonal particles.

Figure 11 illustrates the mechanism of chip formation of SiC p/Al composites in a cutting simulation reinforced by circular particles and irregular polygonal particles, respectively. On the one hand, the reason for the formation of serrated chips is that the addition of reinforced particles increases the brittleness of the material, and plastic deformation and brittle fracture exist simultaneously in the deformation process of SiC p/Al composites. On the other hand, sudden shear occurs during chip formation, originating from the initiation and propagation of microcracks at the particle–matrix interface. As shown in Figure 11a,e, when squeezed by the rake face of the tool, a non-uniform distribution stress that is different from that in homogeneous metal materials is formed near the rake face of the workpiece. When the stress exceeds the interface bonding strength, the interface fails, and microcracks are formed. Bending deformation occurs on the free surface of the workpiece under compression stress (Figure 11b,f). With the advancement of the tool, the microcracks generated by interface debonding in the first deformation zone propagate into large cavities and converge with internal defects of material. Here, there is a huge shear stress along the shear direction, which leads to the sudden shear of chips along the shear direction, forming serrated chips, as illustrated in Figure 11c,d,g,h.

**Figure 12:**
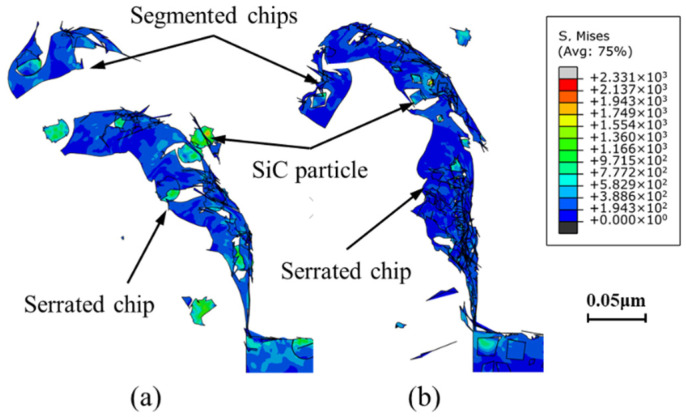
Morphology of chips during cutting simulation: (**a**) circular particles; and (**b**) irregular polygonal particles.

Matrix failure dominates the fracture process during the machining of MMCs reinforced by nano-sized SiC particles [25]. Compared with circular particles under the same volume fraction ratio, the effect of more corners and more serious grain refinement on stress distribution is obvious in the metal matrix reinforced by irregular polygonal particles. The effect of particle refinement leads to a larger number of particle–matrix interfaces and a greater number of propagation paths of cracks, making the crack propagation process smoother. Therefore, in contrast to the smooth serrated chips formed under the circular particle model, sharper serrated chips are obtained under the irregular polygonal particle model. Moreover, particle shape also affects chip morphology. As shown in Figure 12, segmented serrated chips can be obtained with models using particles of different shape. Compared with circular particles, the chip morphology obtained with the irregular polygonal particle model makes it easier to produce discontinuous chips, which may be determined by the presence of serious particle cluster phenomena on the metal matrix under the irregular polygonal particle model. There are less stable particle–matrix interface phases in the area of particle clusters, and it is easier for the cracks around particles to spread to the chip root and separate the chips. Therefore, it can be inferred that the increase in the number of particles results in more serious particle refinement and a less stable particle–matrix interface during the machining of MMCs with a high volume fraction, which is conducive to the extension and propagation of cracks in the shear zone and the generation of segmented chips.

**Figure 13:**
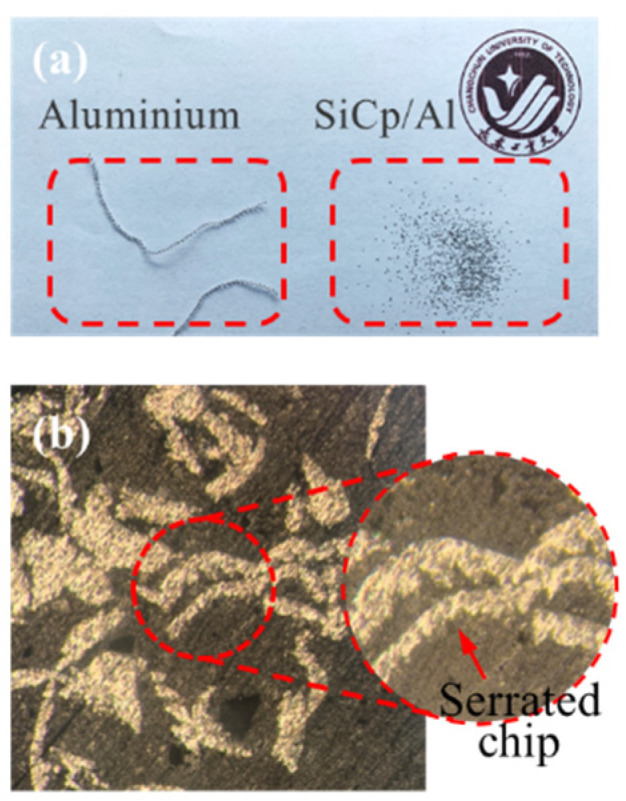
Microstructure of chips under a cutting speed of 250 mm/s and a cutting depth of 25 μm (**a**) aluminum alloy; (**b**) SiC p/Al composites.

Figure 13 presents the chip microstructure under a cutting speed of 250 mm/s and a cutting depth of 25 μm. The results show that the simulation results of the two models are in good agreement with the actual chip morphology. Due to the inhomogeneity and internal micro defects of the material, the initiation and propagation of microcracks cause the instantaneous shear stress and shear area to change constantly during the cutting process, resulting in the formation of serrated chips with quasi-periodic variations in chip thickness. The most obvious difference between aluminum alloy and SiC p/Al composites is the generation of discontinuous (Figure 13a) serrated chips (Figure 13b) during the machining of SiC p/Al composites.

### 4.4. Surface Topography

**Figure 14:**
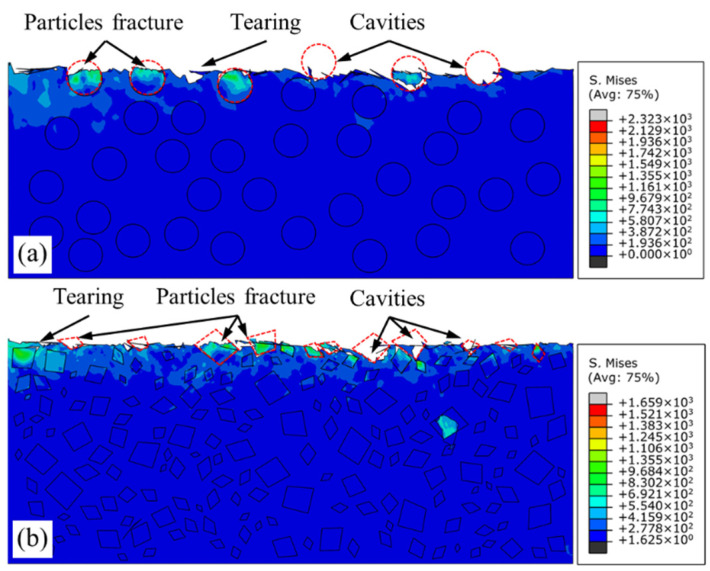
Simulated surface morphology under a cutting speed of 250 mm/s and a cutting depth of 25 μm: (**a**) circular particles; (**b**) irregular polygonal particles.

**Figure 15:**
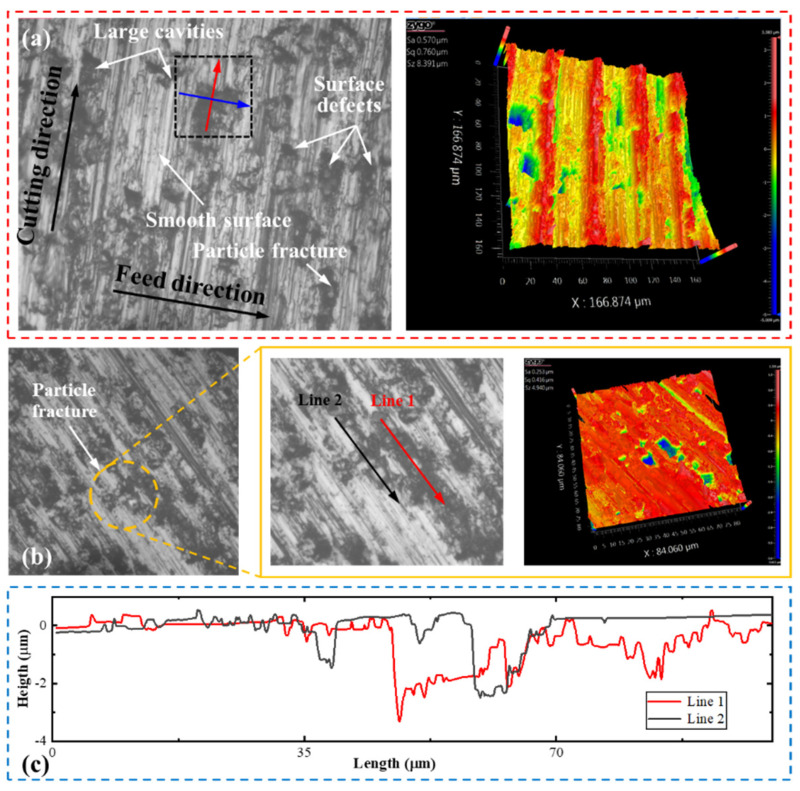
Micrographs of machined surface under a cutting depth of 25 μm and a cutting speed of 250 mm/s. (**a**) surface defects; (**b**) particles embedded in matrix; (**c**) cross section morphology of embedded particles.

The machined surface of the two FE models reinforced using particles with different shapes is mainly dominated by particle fracture, particle–matrix interface debonding, and interaction between tool and workpiece, as illustrated in Figure 14. During the cutting process, SiC particles located on the cutting path fracture under the extrusion of the rake face. Partial particles adhere to the cutting edge and move along the cutting path, causing small scratches on the machined surface, accompanied by matrix tearing. The effect of another partial particles on the machined surface is mainly dependent on the particle–matrix interface, and is mainly related to the relative position of the cutting path and the SiC particles [28]. Large cavities are formed on the machined surface when the fractured particles are pulled out, while the broken particles will be wedged into the metal matrix under the extrusion of the cutting edge and the formation of small pits. In the third deformation zone, the elastic recovery of the metal matrix can cover part of the cavities, optimizing the machined surface quality to a certain extent [41]. The micrographs of the machined surface are shown in Figure 15. Compared with the cutting experiment, the surface morphology predicted by models with differently shaped particles maintain good consistency with the experimental results. The fractured and debonded particles leave pits on the machined surface, which reduces the surface quality, as shown in Figure 15a. Another partial particle is crushed into the chip–rake face interface, causing serious abrasive wear on the tool [42]. In addition, particle fracture causes cavities and deterioration in the surface quality of the machined workpiece. However, some of the fractured particles can be embedded in metal matrix under the ironing pressure of the tool flank face, which smoothes the surface, as shown in Figure 15b,c.

**Figure 16:**
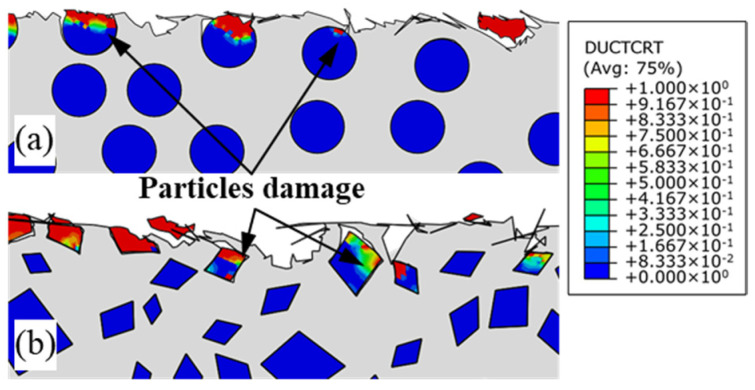
Particle damage of subsurface (**a**) circular particles; (**b**) irregular polygonal particles.

Damage at the integral point of the element can be represented by DUCTCRT in ABAQUS, and Figure 16 shows the subsurface particle damage obtained using the circular and irregular polygonal particle models, respectively. The squeezing action formed at the flank face of the tool is transmitted by the metal matrix to the SiC particles during the cutting process. The damage in the circular particle model is mainly concentrated in parts in contact with or adjacent to the flank face. Meanwhile, the irregular polygonal particle is prone to concentration of stress, and damage is mainly distributed at sharp corners. In addition, a larger vertical component of cutting force can be obtained with the increase of cutting depth. The compressive stress generated by the metal matrix is transferred to the SiC particles, as well as underneath them, aggravating the subsurface damage of the machined surface. Therefore, the irregular polygonal particle model provides a more comprehensive prediction of the subsurface damage of MMCs.

## 5. Conclusions

This paper analyzes the effect of particle shape on deformation mechanism during the cutting simulation of MMCs. Two 2D mesoscopic-based FE models of MMCs reinforced with circular and irregular polygonal particles were developed. The matrix deformation behaviors, distribution and fracture of particles and the interaction at the matrix–particle interfaces were comprehensively considered in the proposed models. Systematic cutting experiments were conducted to evaluate the effect of different particle geometries on the removal mechanism during the simulation process of SiC p/Al composites. The major conclusions of this study were drawn as follows:

(1) Particle fracture is mainly determined by tool–particle interaction, and the metal matrix carries out the role of load transfer. Due to the existence of sharp corners, the stress concentration of irregular polygonal particles is not only affected by the extrusion of the rake face, but is also related to the interaction of the surrounding particles.
(2) The differences in tensile stress and compressive stress between the sides of the particles can explain the cause of the particle damage below cutting path. The particle–matrix interface of the irregular polygonal particle model is not able to uniformly transfer the unbalanced stress on both sides of particle under a trend of particle rotation, which leads to a centrosymmetric stress distribution in the particles. The difference in the speed of plastic deformation between both sides of the particles above the cutting path accounts for the particle rotation in the chips.
(3) The two models reinforced by circular and irregular polygonal particles were able to effectively predict variations in the cutting force, with large fluctuations and small fluctuations also appearing in the cutting force curve. Due to the dynamometer potentially not being able to capture all impacts caused by each of the SiC particles in the cutting test, the irregular polygonal particle model is more reasonable for reflecting changes in the cutting force in the actual cutting process of SiC p/Al composites than the circular particle model.
(4) The formation, propagation and convergence of microcracks have significant impacts on chip formation and morphology, particle fracture and debonding, and surface integrity. The interface stability is poor in the irregular polygonal particle model, which facilitates the formation of sharper serrated chips.
(5) The machined surface of SiC/Al composites is mainly dominated by particle fracture, particle–matrix interface debonding, and interaction between tool and workpiece. Considering the actual microstructure of MMCs, the irregular polygonal particle model provides a more comprehensive prediction of subsurface damage.

This paper provides guidance for the finite element model of MMCs, and helps to improve the accuracy of the cutting simulation of composites. However, the cutting of MMCs is a complex deformation process. In addition to the cutting mechanism analyzed above, other influencing parameters include cutting parameters, tool material and wear (built-up edge), as well as the deformation mechanism affected by the manufacturing process of the material itself. Therefore, more factors will be considered in future research.

## Figures and Tables

**Table 1:** Machining parameters and tool specifications.

Parameters	Values
Cutting speed (mm/s)	250
Cutting depth (mm)	0.025
Feed rate (mm/rev)	0.01
Tool material	PCD
Rake angle (°)	7
Flank angle (°)	5
Particle size (μm)	15 (5–20)
Volume fraction (%)	25

**Table 2:** Material properties and Johnson–Cook (JC) constants of the Al matrix.

Parameters	Values
Density (ton/mm^3^)	2820 × 10^−12^
Young’s Modulus (MPa)	70,600
Poisson’s Ratio	0.35
*A* (MPa)	224
*B* (MPa)	426
*C*	0.003
*n*	0.2
*D*_1_	0.13
*D*_2_	0.13
*D*_3_	−1.5
*D*_4_	0.011

**Table 3:** Material properties and constants of SiC particles.

Parameters	Values
Density (ton/mm^3^)	3200 × 10^−12^
Young’s Modulus (MPa)	408,000
Poisson’s Ratio	0.35
Tensile Strength (MPa)	2000
Friction angle (°)	13
Expansion angle (°)	−5
*k*	0.92
*G* (MPa)	1000
*p*	2
$e_{\max}^{ck}$	0.2

**Table 4:** Specific parameters of interface properties.

Parameters	Values
Fracture energy (J/m^2^)	50
Elastic moduli (MPa)	180,600
Shear moduli (MPa)	76,600
Interfacial strength (MPa)	372

## Data Availability

All data needed to evaluate the conclusions in the paper are present in the paper. Additional data related to this paper may be requested from the authors.
